# Painless Tooth Extraction

**Published:** 1896-04

**Authors:** 


### Painless Tooth Extraction.

| | | |
|---|---|---|
| ℞ | Oil wintergreen | 2 drachms. |
| | Chloroform | 1 drachm. |
| | Sulphuric ether | 1 drachm. |
| | Chloral hydrat | 2 drachms. |
| | Oil of cloves | 4 drachms. |
| | Alcohol | 12 drachms. |

M. Apply with cotton, pressed on each side of the tooth.—*Medical Standard.*

THE writings upon antiseptics are multiple, and their appearance ever and anon has become monotonous to many of us, yet there are operators to-day who fail utterly to observe one law of antisepsis. I visited a dental office recently where the operator disregards the teachings of cleanliness to a painful extreme. Several patients called during my stay, and each one was met with the same unsterilized instrument. Put yourself in their place, how would you like such shameful treatment? Is not this an argument in favor of antiseptics?—DR. D. E. WIBER *in Medical Brief.*

